# Effects of proton radiation on evoked and spontaneous neuronal activity in the hippocampus of APP/PSEN1 transgenic mice

**DOI:** 10.1093/jrr/rrt174

**Published:** 2014-03

**Authors:** Emil Rudobeck, Attila Szucs, Roman Vlkolinsky

**Affiliations:** 1 Division of Radiation Research, Department of Basic Sciences, 11175 Campus Street, Chan-Shun Pavilion, 1010A, Loma Linda University, Loma Linda, CA 92350, USA; 2 BioCircuits Institute, University of California San Diego, La Jolla, CA, USA

**Keywords:** radiation, hippocampus, synaptic transmission, electrophysiology

## Abstract

Ground-based studies on space radiation indicate that exposure to charged particle radiation at relatively low doses may impair neuronal functions. Decrements in excitatory synaptic transmission and plasticity in the hippocampus have been previously reported in mouse brains irradiated with iron nuclei, but relatively little is known about the effects of protons on the synaptic activity in the hippocampus. Behavioral and neurophysiological decrements in irradiated subjects are also commonly observed in Alzheimer's disease (AD), and we hypothesized that irradiation with protons may exacerbate AD-like neurodegenerative pathology. We used transgenic (TG) mice with AD-like neurodegeneration to test whether whole-body irradiation with protons (150 MeV; 0.1, 0.5, 1 Gy) accelerates the onset of AD and/or exacerbates synaptic impairments. We measured evoked excitatory synaptic potentials, synaptic plasticity and spontaneous oscillations in hippocampal slices prepared from APP/PSEN1 double TG mice (males only) at 6 and 9 months post-irradiation.

Our electrophysiological recordings indicate that most of the radiation-induced synaptic decrements can be observed at 9 months rather than 6 months post-irradiation and they are differently expressed in TG and wild-type (WT) mice. Presynaptic excitability evaluated by the amplitude of fiber volleys was significantly increased in non-irradiated TG mice when compared with non-irradiated WT mice, and irradiation did not cause further alterations. The post-synaptic, dendritic excitability, evaluated by the initial slope of the excitatory post-synaptic potentials (EPSPs), was reduced in TG mice exposed to 0.5 Gy, but increased relative to WT mice at the same dose. Interestingly, the suppressive effect on EPSP in TG mice was not observed at 1 Gy. Post-synaptic firing of action potentials, evaluated by the amplitude of population spikes (PS), was increased in non-irradiated TG mice when compared with WT controls. However, these increases were significantly reduced by irradiation at 0.1 and 1 Gy. Long-term potentiation of the EPSPs, a widely used electrophysiological correlate of memory formation, was not significantly affected by irradiation. Last, in WT mice, we observed a significant radiation-induced decrease in the frequency of epileptiform oscillations in the CA3–CA1 network triggered by reduced concentration of extracellular magnesium. These spontaneous waveforms (SW) are reminiscent of ‘sharp-wave/ripple complexes’ that have been associated with memory consolidation in the hippocampus. Interestingly, in the behavioral part of our project, irradiation impaired hippocampal learning in WT mice, while it did not affect performance in TG mice [ 1]. This may indicate altered memory consolidation in irradiated WT mice, where we observed significant negative correlation between SW and increased swim distance in water maze. Our data indicate that irradiation with protons at low doses may differently affect synaptic parameters in WT and APP/PSEN1 TG mice. Thus, different neurological or cognitive decrements may be expected in normal subjects relative to those prone to AD-like pathology. The most prominent radiation-induced decrements in TG mice were reduced dendritic excitability and firing of CA1 neurons. The reduction in PS amplitudes in TG mice suggests that proton radiation may impair communication of the hippocampal neurons with other brain areas. Memory formation and consolidation processes in TG mice are likely not further affected by the irradiation.
